# FAIR for AI: An interdisciplinary and international community building perspective

**DOI:** 10.1038/s41597-023-02298-6

**Published:** 2023-07-26

**Authors:** E. A. Huerta, Ben Blaiszik, L. Catherine Brinson, Kristofer E. Bouchard, Daniel Diaz, Caterina Doglioni, Javier M. Duarte, Murali Emani, Ian Foster, Geoffrey Fox, Philip Harris, Lukas Heinrich, Shantenu Jha, Daniel S. Katz, Volodymyr Kindratenko, Christine R. Kirkpatrick, Kati Lassila-Perini, Ravi K. Madduri, Mark S. Neubauer, Fotis E. Psomopoulos, Avik Roy, Oliver Rübel, Zhizhen Zhao, Ruike Zhu

**Affiliations:** 1 Data Science and Learning Division, Argonne National Laboratory, Lemont, Illinois 60439 USA; 2 Department of Computer Science, University of Chicago, Chicago, Illinois 60637 USA; 3 Globus, University of Chicago, Chicago, Illinois 60637 USA; 4 Department of Mechanical Engineering and Materials Science, Duke University, Durham, North Carolina 27708 USA; 5 Scientific Data Division, Lawrence Berkeley National Laboratory, Berkeley, CA 94720 USA; 6 Biological Systems & Engineering, Lawrence Berkeley National Laboratory, Berkeley, California 94720 USA; 7 Helen Wills Neuroscience Institute, University of California Berkeley, Berkeley, California 94720 USA; 8 Department of Physics, University of California San Diego, La Jolla, California 92093 USA; 9 Lund University, Department of Physics, Box 118, 221 00 Lund, Sweden; 10 School of Physics & Astronomy, The University of Manchester, Manchester, M13 9PL UK; 11 Leadership Computing Facility, Argonne National Laboratory, Lemont, Illinois 60439 USA; 12 Biocomplexity Institute and Department of Computer Science, University of Virginia, Charlottesville, Virginia 22904 USA; 13 Department of Physics, Massachusetts Institute of Technology, Cambridge, Massachusetts 02139 USA; 14 Technical University Munich, Arcisstraβe 21, 80333 München, Germany; 15 Computational Science Initiative Brookhaven National Laboratory Upton, New York, 11973 USA; 16 Electrical and Computer Engineering, Rutgers, The State University of New Jersey, Piscataway, New Jersey 08854 USA; 17 National Center for Supercomputing Applications, University of Illinois, Urbana-Champaign, Urbana, Illinois 61801 USA; 18 Department of Computer Science, University of Illinois at Urbana-Champaign, Urbana, Illinois 61801 USA; 19 Department of Electrical & Computer Engineering, University of Illinois at Urbana-Champaign, Urbana, Illinois 61801 USA; 20 School of Information Sciences, University of Illinois at Urbana-Champaign, Urbana, Illinois 61801 USA; 21 San Diego Supercomputer Center, University of California San Diego, La Jolla, California 92093 USA; 22 Helsinki Institute of Physics, University of Helsinki, P.O. Box 64, Helsinki, 00014 Finland; 23 Department of Physics, University of Illinois at Urbana-Champaign, Urbana, Illinois 61801 USA; 24 Institute of Applied Biosciences, Centre for Research and Technology Hellas, Thessaloniki, 57001 Greece

**Keywords:** Research management, Research data

## Abstract

A foundational set of findable, accessible, interoperable, and reusable (FAIR) principles were proposed in 2016 as prerequisites for proper data management and stewardship, with the goal of enabling the reusability of scholarly data. The principles were also meant to apply to other digital assets, at a high level, and over time, the FAIR guiding principles have been re-interpreted or extended to include the software, tools, algorithms, and workflows that produce data. FAIR principles are now being adapted in the context of AI models and datasets. Here, we present the perspectives, vision, and experiences of researchers from different countries, disciplines, and backgrounds who are leading the definition and adoption of FAIR principles in their communities of practice, and discuss outcomes that may result from pursuing and incentivizing FAIR AI research. The material for this report builds on the FAIR for AI Workshop held at Argonne National Laboratory on June 7, 2022.

## Introduction

The production, collection, and curation of data require painstaking planning and the use of sophisticated experimental and computational facilities. In order to maximize the impact of these investments and create best practices that lead to scientific discovery and innovation, a diverse set of stakeholders defined a set of findable, accessible, interoperable, and reusable (FAIR) principles in 2016^1,2^. The original intent was that these principles would apply seamlessly to data and all scholarly digital objects, including research software^3^, workflows^4^, and even domain-specific custom digital objects^5^. However, because they were specifically written in the context of data, it became clear over time that the original set of FAIR principles would have to be translated or reinterpreted for digital assets beyond data^6,7^. This realization has led to initiatives that have proposed and/or developed practical FAIR definitions for research software and workflows, and more recently, for artificial intelligence (AI) models^8,9^.

In this document, we provide an inclusive and diverse perspective of FAIR initiatives in Europe and the US through the lens of researchers that are leading the definition, implementation, and adoption of FAIR principles in a variety of disciplines. This community was brought together at the FAIR for AI Workshop (https://indico.cern.ch/event/1152431/) at Argonne National Laboratory on June 7, 2022. We believe that this document provides a factual, straightforward, and inspiring description of what FAIR initiatives have accomplished, what is being done and planned at the time of writing this document, and describes the end goals of these disparate initiatives. Most importantly, we hope that the ideas presented in this document serve as a motivator to reach convergence on what FAIR means, in practice, for AI research and innovation.

## FAIR Initiatives

We have identified the following non-exhaustive list of FAIR initiatives:

- FAIR4HEP: Findable, Accessible, Interoperable, and Reusable Frameworks for Physics-Inspired Artificial Intelligence in High Energy Physics (https://fair4hep.github.io). Funded by the US Department of Energy (DOE). In this project an interdisciplinary team of physicists, computer, and AI scientists use high energy physics as the science driver to develop a FAIR framework that advances our understanding of AI, provides new insights to apply AI techniques, and provides an environment where novel approaches to AI can be explored.
- ENDURABLE: Benchmark Datasets and AI models with queryable metadata (https://sites.google.com/lbl.gov/endurable/home). Funded by DOE. The goal of this project is to provide the scientific and machine learning (ML) communities with robust, scalable, and extensible tools to share and rigorously aggregate diverse scientific data sets for training state-of-the-art ML models.
- The Common Fund Data Ecosystem (https://commonfund.nih.gov/dataecosystem). Funded by the US National Institutes of Health (NIH). An online discovery platform (https://app.nih-cfde.org) that enables researchers to create and search across FAIR datasets to ask scientific and clinical questions from a single access point.
- BioDataCatalyst (https://biodatacatalyst.nhlbi.nih.gov). Funded by NIH. Construct and enhance annotated metadata for heart, lung, and blood datasets that comply with FAIR data principles.
- Garden: A FAIR Framework for Publishing and Applying AI Models for Translational Research in Science, Engineering, Education, and Industry (https://thegardens.ai). Funded by the US National Science Foundation (NSF). This project will reduce barriers to the use of AI methods and promote the nucleation of communities around specific FAIR datasets, methods, and AI models. Model Gardens will provide a repository for models where they can be linked to papers, testing metrics, known model limitations, and code, plus computing and data storage resources through tools such as the Data and Learning Hub for Science^10^, funcX^11^ and Globus^12^.
- Braid: Data Flow Automation for Scalable and FAIR Science (https://anl-braid.github.io/braid/). Funded by DOE. This project aims to enable researchers to define sets of flows that individually and collectively implement application capabilities while satisfying requirements for rapid response, high reconstruction fidelity, data enhancement, data preservation, model training, etc.
- HPC-FAIR: A Framework Managing Data and AI Models for Analyzing and Optimizing Scientific Applications (https://hpc-fair.github.io/). Funded by DOE. This multi-institutional project aims to develop a generic High Performance Computing data management framework^13,14^ to make both training data and AI models of scientific applications FAIR.
- The FAIR Surrogate Benchmarks Initiative (https://sbi-fair.github.io). Funded by DOE. The research develops AI surrogates and studies their key features and the software environment to support their use^15^ in simulation based research. They collaborate with MLCommons (https://mlcommons.org/en/), a consortium including 62 companies that host the MLPerf benchmarks, including those for science^16–18^, and mirror their processes in the computational science domain. This involves rich metadata involving models, datasets, and the logging of their use with machine and power characteristics recorded, requiring multiple ontologies to be developed with FAIR approaches.
- The Materials Data Facility (MDF) (https://www.materialsdatafacility.org). Funded by the National Institute of Standards and Technology (NIST) and the Center for Hierarchical Materials Design, the MDF^19,20^ aims at making materials data easily publishable, discoverable, and reusable while following and building upon the FAIR principles. To date, MDF has collected over 80 TB of materials data in nearly 1000 datasets. In particular, this effort enables publication of datasets with millions of files or datasets comprising TB of data, and seeks to automatically index the contents in ways that provide unique queryable interfaces to the datasets. Recently, these capabilities have been augmented via the Foundry (https://github.com/MLMI2-CSSI/foundry) to provide access to well-described ML-ready datasets with just a few lines of Python code.
- Neurodata Without Borders (NWB) (https://www.nwb.org/). Funded by the NIH BRAIN Initiative. NWB is an interdisciplinary project to create a FAIR data standard for neurophysiology, providing neuroscientists with a common standard to share, archive, use, and build common analysis tools for neurophysiology data. More than just a data standard, NWB is at the heart of a growing software ecosystem for neurophysiology data, including data from intracellular and extracellular electrophysiology experiments, data from optical physiology experiments, and tracking and stimulus data. A growing number of neurophysiology data generated by NIH BRAIN Initiative research projects and others are available on the DANDI neurophysiology data archive.
- Materials Research Data Alliance (MaRDA) (https://www.marda-alliance.org). MaRDA is an organization dedicated to helping to build community to promote open, accessible and interoperable data in materials science. MaRDA has held two virtual workshops reaching 300 attendees last year, and has helped researchers form independent working groups. In August 2022, MaRDA leadership was funded via the NSF Research Coordination Network program to significantly expand efforts to build a sustainable community around these topics, to build consensus in metadata requirements, to train next generation workforce in ML/AI for materials, to develop shared community benchmark challenges, to host convening and coordination events, and more.
- PUNCH4NFDI (https://www.punch4nfdi.de) is the German National Research Data Infrastructure consortium of particle, astro, astroparticle, hadron, and nuclear physics, representing about 9,000 scientists with a PhD in Germany from universities, the Max Planck Society, the Leibniz Association, and the Helmholtz Association. The prime goal of PUNCH4NFDI is to set up a federated and FAIR science data platform, offering the infrastructures and interfaces necessary for access to and use of data and computing resources of the involved communities and beyond.
- ESCAPE (https://www.projectescape.eu) is the European Science Cluster of Astronomy & Particle Physics ESFRI Research Infrastructures, funded from the European Union’s Horizon 2020 research and innovation programme. The goal of ESCAPE is to address the critical questions of open science and long term reuse of data for science and for innovation, many of the greatest European scientific facilities in physics and astronomy have combined forces to make their data and software interoperable and open, committing to make the European Science Cloud a reality. ESCAPE is delivering two Science Projects to aid the prototyping the European Open Science Cloud (EOSC) within the EOSC-Future (https://eoscfuture.eu), another Horizon 2020-funded project. These Science Projects will advance the science, the FAIR data and the software tools needed for dark matter searches and multi-messenger astronomy for extreme universe phenomena such as gravitational waves.
- Awesome Materials Informatics (https://github.com/tilde-lab/awesome-materials-informatics) is an interdisciplinary and community building effort to assemble a list of a holistic set of tools and best practices for Materials Science, encompassing software and products, cloud simulation platforms, and standardization initiatives.

These initiatives suggest that researchers are developing methods, approaches, and tools from scratch to address specific needs in their communities of practice. Thus, it is timely and important to identify common needs and gaps in disparate disciplines, abstract them and then create commodity, generic tools that address similar challenges across fields. Interdisciplinary efforts of this nature may leverage work led by several research data consortia which tend to be more general, e.g., the Research Data Alliance (RDA) (https://www.rd-alliance.org), the International Science Council’s Committee on Data (CODATA) (https://codata.org/), and GO FAIR (https://www.go-fair.org/). This translational approach has been showcased in the context of scientific datasets^21^ and for AI models and datasets^8^. These recent efforts pose an important question: what is the optimal composition of interdisciplinary teams that may work together to create sufficiently generic solutions that may then be specialized down to specific disciplines and projects? As these interdisciplinary teams are assembled, and they work to define, implement, and then showcase how to adopt FAIR principles, it is critical to keep in mind that FAIR is not the goal *per se*, rather the science and innovation that such principles and best practices will enable. As well, FAIR is not a goal as much as a continual process.

In high energy physics (HEP), the experiments at the Large Hadron Collider at CERN are committed to bringing their data into the public domain^22^ through the CERN Open Data portal (http://opendata.cern.ch/). The CMS experiment has led the effort and, since 2014, made close to 3PB of research-level data public. Their availability opens unprecedented opportunities to process samples from original HEP experiment data for different AI studies. While the experiment data distribution follows FAIR principles, they remain complex, and their practical reusability has required further thoughts on the FAIR principles concretely applicable to software and workflows. Furthermore, the application of FAIR principles to data and AI models is important for the sustainability of HEP science and enhancing collaborative efforts with others, both inside and outside of the HEP domain. Ensuring that data and AI models are FAIR facilitates a better understanding of their content and context, enabling more transparent provenance and reproducibility^23,24^. There is a strong connection between FAIRness and interpretability, as FAIR models facilitate comparisons of benchmark results across models^25^ and applications of *post-hoc* explainable AI methods^26^. As described in ref. ^27^ data and AI models preserved in accordance with FAIR principles can facilitate education in data science and machine learning in several ways, such as interpretability of AI models, uncertainty quantification, and ease of access of data and models for key HEP use cases. In this way, they can be reliably reused to reproduce benchmark results for both research and pedagogical purposes. For instance, the detailed analysis of FAIR and AI-readiness of the CMS $H(b\bar{b})$ dataset in ref. ^21^ has explained how the FAIR readiness of this dataset has been useful in building ML exercises for open source courses on AI for HEP^28^.

In the materials science domain, the importance of broad accessibility of research data on all materials and the transformative potential impact of FAIR data and use of data driven and AI approaches was recognized with the advent of the Materials Genome Initiative (MGI) in 2011^29^, and with a recently released MGI Strategic Plan in late 2021^30^. In the decade since the launch of MGI, the power of integrating data science with materials science has unleashed an explosion of productivity^31,32^. Early adopters were computational materials scientists who launched a number of accessible data portals for hard materials and who have begun working together across the world on interoperability standards^33^. Subsequently, significant efforts have been launched towards capturing FAIR experimental data and tackling the complexities of soft materials^34^. In the last several years, MaRDA has developed and flourished with multiple workshops and working groups addressing issues of FAIR data and models across all aspects of materials science.

In the life sciences, AI is becoming increasingly popular as an efficient mechanism to extract knowledge and new insights from the vast amounts of data that are constantly generated. AI has the potential for transformative impact on the life sciences since almost half of global life sciences professionals are either using, or are interested in using, AI in some area of their work^35^. This transition is clearly shown in the explosion of ML articles in life sciences over the past decade: from around 500 such publications in 2010 to approximately 14k publications in 2020, an exponential increase that does not show any signs of slowing down in the short-term^36^. However, AI is not a one-solution-fits-all, nor a magic wand that can address any challenge in the life sciences and beyond. In this context, scientists pursuing domain aware AI applications may benefit from defining community-backed standards, such as the DOME recommendations^36^, which were spearheaded by the ELIXIR infrastructure (https://elixir-europe.org/). As scientists adopt these guidelines, and prioritize openness in all aspects of their work processes, FAIR AI research will streamline the creation of AI applications that are trustworthy, high quality, reliable, and reproducible.

### Towards a practical definition of FAIR for AI models

There are several efforts that aim to define, at a practical level, what FAIR means for scientific datasets and AI models. As a starting point, researchers have created platforms that provide, in an integrated and centralized manner, access to popular AI models and standardized datasets, e.g., the Hugging Face (https://huggingface.co) platform, and the Data and Learning Hub for Science^10^.

While these efforts are necessary and valuable, additional work is needed to leverage these AI models and datasets, and translate them for AI R&D in scientific applications. This is because state-of-the-art AI models become valuable tools for scientific discovery when they encode domain knowledge, and are capable of learning complex features and patterns in experimental datasets, which differ vastly from standardized datasets (ImageNet, Google’s Open Images, xView, etc.). Creating scientific AI tools requires significant investments to produce, collect and curate experimental datasets, and then incorporate domain knowledge in the design, training and optimization of AI models. Often, this requires the development and deployment of distributed training algorithms in high performance computing (HPC) platforms to reduce time-to-insight^37,38^, and the optimization of fully trained AI models for accelerated inference on HPC platforms and/or at the edge^39,40^. How can this wealth of knowledge be leveraged, extended or seamlessly used by other researchers that face similar challenges in similar or disparate disciplines?

While peer-reviewed publications continue to be the main avenue to communicate advances in AI for science, researchers increasingly recognize that articles should also be linked to data, AI models, and scientific software needed to reproduce and validate data-driven scientific discovery. Doing so is in line with the norm in scientific machine learning, which is characterized by open access to state-of-the-art AI models and standardized datasets. This is one of the central aims in the creation of FAIR datasets and AI models, namely, to share knowledge, resources, and tools following best practices to accelerate and sustain discovery and innovation.

Several challenges, however, need to be addressed when researchers try to define, implement and adopt FAIR principles in practice. This is because there is a dearth of simple-to-follow guidelines and examples, and of lack of consistent metrics that indicate when the FAIRification of datasets and AI models has been done well or not, and how to improve. Furthermore, while the FAIR principles are simple to read, they can be difficult to implement, and work is needed to build consensus about what they mean in specific cases, how they can be met, and how implementation can be measured, not only for data but also for other types of digital objects, such as AI models and software. The need to integrate FAIR mechanisms throughout the research lifecycle has been noted^41^. Researchers are actively trying to address these gaps and needs in the context of datasets^21^ and AI models.

On the latter point, two recent studies^8,9^ have presented practical FAIR guidelines for AI models. Common themes in these studies encompass: 1) the need to define the realm of applicability of these principles in the AI R&D cycle, i.e., they consider AI models that have been fully trained and whose FAIRness is quantified for AI-driven inference; 2) the use of common software templates to develop and publish AI models, e.g., the template generator cookiecutter4fair^42^; and 3) the use of modern computing environments and scientific data infrastructure to transcend barriers in hardware architectures and software to speak a common AI language. To ground these ideas, refs. ^8,9^ proposed definitions of a (FAIR) AI model, which we have slightly modified as follows: “an AI model comprises a computational graph and a set of parameters that can be expressed as scientific software that, combined with modern computing environments, may be used to extract knowledge or insights from experimental or synthetic datasets that describe processes, systems, etc. An AI model is Findable when a digital object identifier (DOI) can direct a human or machine to a digital resource that contains the model, its metadata, instructions to run the model on a data sample, and uncertainty quantification metrics to evaluate the soundness of AI predictions; it is Accessible when it and its metadata may be readily downloaded or invoked by humans or machines via standardized protocols to run inference on data samples; it is Interoprable when it can seamlessly interact with other models, data, software, and hardware architectures; and it is Reusable when it can be used by humans, machines and other models to reproduce its expected inference capabiblities, and provide reliable uncertainty quantification metrics when processing datasets that differ from those originally used to create it and quantify its performance”.

Furthermore, the work presented by Ravi *et al*.^8^, emphasizes the need to create computational frameworks that link FAIR and AI-ready datasets (produced by scientific facilities or large scale simulations and either hosted at data facilities or broadcast to supercomputing centers) with FAIR AI models (hosted at model hubs), and that can leverage computing environments (e.g., supercomputers, AI-accelerator machines, edge computing devices, and the cloud) to automate data management and scientific discovery. All these elements may be orchestrated and steered by Globus workflows.

### Rationale to invest in FAIR research

There are many compelling reasons to create and share FAIR AI models and datasets. Recent studies argue that FAIR data practices are not only part of good research practices, but will save research teams time by decreasing the need for data cleanup and preparation^43^. It is easy to dismiss anything that sounds new as an “unfunded mandate”. However, FAIR directly relates to many compatible initiatives and goals of most scientifically focused organizations. For instance, FAIRness is closely connected to, and perhaps a prerequisite of, reproducibility. It is also needed for data exploration and is closely connected to ethics issues. FAIR principles can contribute to transparency and other tenets of Open Science.

On the other hand, Supercomputing resources (e.g., Argonne Leadership Computing Facility, Oak Ridge Leadership Computing Facility, National Center for Supercomputing Applications, Texas Advanced Computing Center, etc.,) and scientific data facilities (e.g., Advanced Photon Source at Argonne, National Synchrotron Light Source II at Brookhaven National Laboratory, etc.,) produce valuable data that may only be effectively shared and reused through the adoption of practical, easy to follow FAIR principles, and the design and deployment of smart software infrastructure. In brief, FAIR is an important step towards an optimal use of taxpayer dollars, it maximizes the science reach of large scale scientific and cyberinfrastructure facilities to power automated AI-driven discovery.

### Needs and gaps in AI research that may be addressed by adopting FAIR principles

In the article that established the FAIR Principles, it is emphasized these principles should enable machine actionable data^44^. This is synergistic with the rapid adoption and increased use of AI in research. The more data is easy to locate (**F**indable), easy to access (**A**), well described with good and interoperable metadata (**I**), and available for reuse (**R**) the easier it will be to use existing data as training or validation sets for AI models. Specific benefits of FAIR AI research throughout the entire discovery cycle include:

- Rapid discovery of data via search and visualization tools, ability to download data for benchmarking and meta-analyses using AI for further scientific discovery.
- Reproducibility of papers and AI models published with them.
- Easy-to-follow guides for how to make data and AI models FAIR are needed, as this process can be difficult, particularly for researchers to whom it is new.
- Establish and promote tools and data infrastructures that accept, store, and offer FAIR and AI-ready data.
- In biomedicine and healthcare, AI models could improve generalization by exposing them to diverse, FAIR datasets.
- Engagement from industry partners is vital to this effort, since they are a major force in AI innovation.
- Get publishers involved and committed to using FAIR, both for data and for other objects such as AI models and software, as they are where research results are shared.
- Adopting the FAIR principles in AI research will also facilitate more effective reporting. Inadequate explanations on the main parts of AI methods not only lead to distrust of the results, but also act as blockers in transferring them to an applied context, such as the clinic and patient care.
- Making FAIR datasets available in HEP is crucial to obtaining benchmark performances of AI models that make AI-driven discovery possible. While a large number of models have been developed for targeted tasks like classification of jets in collider experiments^45^, their performances vary with the choice of training datasets, their preprocessing, and training conditions. Developing FAIR datasets and FAIRifying AI models with well defined hyperstructure and training conditions will allow uniform comparison of these models.
- Establishing seamless and interoperable data e-infrastructures. As these infrastructures mature, a new *AI services layer* will emerge; defining the FAIR principles in advance is thus important in order to accelerate this process.
- Computer science and AI research on efficient generic surrogate architectures and methods to derive reliable surrogate performance for a given accuracy (i.e., towards general surrogate performance models) will benefit extensively from FAIR data and processes.
- One element that has been often debated in AI solutions is of fair (or unbiased) models. This issue is one of the most critical in life sciences, and especially when considering applications that have a direct consequence to human health. FAIR AI and data can facilitate the overall process of identifying potential biases in the involved process.
- Where reproducibility cannot be guaranteed, FAIR data and processes can help establish at a minimum scientific correctness.

### Agreed-upon approaches/best practices to identify foundational connections between scientific (meta)datasets, AI models, and hardware

Since this work is in its infancy, there is an urgent need to create incentive structures to impel researchers to invest time and effort to adopt FAIR principles in their research, since these activities will lower the barrier to adopting AI methodologies. Adopting FAIR best practices will bring about immediate benefits. For instance, FAIR AI models can be constantly reviewed and improved by researchers. Furthermore, software can be optimized for performance or expanded in functionality, rather than standing still and stagnant. In materials science and chemistry, and many other disciplines, thousands of AI models are published each year. Thus, it is critical to rank best AI models, FAIRly share them, and develop APIs to streamline their use within minutes or seconds. Specific initiatives to address these needs encompass:

- GOFAIRUS (https://www.gofair.us). FAIR papers that efficiently link publications, AI models, and benchmarks to produce figures of merit that quantify performance of AI models and sanity of datasets.
- MLCommons (https://mlcommons.org/en/). A consortia that brings industry and academic partners together in a pre-competitive space to compare performance of specific tasks and datasets using different hardware architectures and software/hardware combinations.
- Garden (https://thegardens.ai). A platform for publishing, discovering and resuing FAIR AI models, linked to FAIR and AI-ready datasets, in physics, chemistry and materials science.
- Bridge2AI (https://commonfund.nih.gov/bridge2ai). FAIR principles can enable ethics inquiries in datasets, easing their use by communities of practice.

While these approaches aim to ease the adoption and development of AI models for scientific discovery and to develop methods to quantify the statistical validity, reliability and reproducibility of AI for inference, there are other lines of research that explore the interplay between datasets, AI models, optimization methods, hardware architectures, and computing approaches from training through to inference. It is expected that FAIR and AI-ready datasets may facilitate these studies. For instance, scientific visualization and accelerated computing have been combined to quantify the impact of multi-modal datasets to optimize the performance of AI models for healthcare^46^, cosmology^47,48^, high energy physics^9,49^, and observational astronomy^50,51^, to mention a few exemplars. These studies shed new light into the features and patterns that AI extracts from data to make reliable predictions. Similarly, recent studies^52–54^ have demonstrated that incorporating domain knowledge in the architecture of AI models, and optimization methods (through geometric deep learning and domain aware loss functions) leads to faster (even zero shot) learning and convergence, and optimal performance with smaller training and validation datasets.

It is also worth mentioning that publishing a FAIR AI model including all relevant (meta)data, e.g., set of initial weights for training, all relevant hyperparameters, libraries, dependencies, and the software needed for training and optimization may not suffice to attain full reproducibility. This is because users may use different hardware to train and optimize AI models, and thus the selection of batchsize and learning rate may have to be adjusted if only one or many GPUs are used for distributed training. It may also be the case that users prefer to use AI-accelerator machines and the AI model, hyperparameters, libraries and dependencies will have to be changed. These considerations have persuaded researchers to define FAIRness in the context of AI inference. These caveats were also discussed by Ravi *et al*.^8^, where a FAIR AI model was produced using distributed computing with GPUs, quantized with NVIDIA TensorRT, and trained from the ground up using the SambaNova DataScale system at the ALCF AI Testbed (https://www.alcf.anl.gov/alcf-ai-testbed). However, FAIRness of these different AI models was quantified at the inference stage.

### Promise or role of privacy preserving and federated learning in the creation of FAIR AI datasets and models

Sample case: PALISADE-X Project (https://www.palisadex.net). The scope of applications in this project includes development of AI models using closed source/sensitive data and leveraging distributed secure enclaves. Current applications include biomedical data, but may be applicable to data from smart grids, national security, physics, astronomy, etc.

The development of FAIR AI tools for privacy preserving federated learning should be guided by several considerations. For instance, ethically sourced data (beyond human safety protection) should include attributes for the creation of AI models in a responsible manner. Furthermore, open, AI-driven discovery with protected data should be guided with clear principles and examples that demonstrate how to use data in a way that protects the privacy of individuals or organization. Ethical data sharing and automated AI-inference results should be regulated with input from interdisciplinary teams. Care should be taken to perform a thorough external validation of developed models to capture diversity and measure their applicability across different data distributions. In the case of personalized medicine, existing smart watches can identify markers that may identify suicidal behaviour. Should these results be readily shared with healthcare provider without input from individuals? These considerations demand thoughtful policy development and governance for datasets and AI models.

Ethical issues go well beyond biology, genomics and healthcare. For instance, in materials science and chemistry a recent article described a methodology to train an AI model to minimize drug toxicity, and then used to show potential misuse of maximizing toxicity for chemical weapons development^55^.

### Transparent/interpretable AI models are considered critical to facilitating the adoption of AI-driven discovery. Why is (or isn’t) this possible/reasonable in view of the ever increasing complexity of AI models?

AI models have surpassed human performance in image classification challenges^56,57^. These algorithms process data, identify patterns and features in different ways to humans. When we try to understand what these AI models learn and how they make decisions, we should avoid using human-centric judgements on what is correct or acceptable. These algorithms need not work or “think” as humans to be promoted as reliable and trustworthy tools for scientific discovery and innovation. Rather, we should focus on defining clear, easy to follow, quantifiable principles to thoroughly examine AI predictions. At the same time, it is important to distinguish persuasive^58^ from interpretable AI^59^.

Scientific visualization is a powerful tool to explore and get new insights on how and what AI models learn; the interplay among data, a model’s architecture, training and optimization schemes (when they incorporates domain knowledge) and hardware used; and what triggers a sharp response in an AI model that is related to new phenomena or unusual noise anomalies^47,48^.

Explainability of AI models can be deemed crucial in scientific domains when the decision making process of deep learning models can be important to make them trustworthy and generalizable. Interpretability of deep neural networks is important to identify relative importance of features and identify information pathways within the network. With prohibitively large complexities of neural architectures, existing methods of explainable AI can be constrained by their lack of scalability and robustness. Domain specific approaches for developing novel methods in explainable AI need to be explored to ensure development of reliable and reusable AI models^26,60^.

A number of strategies to create explainable AI models include the use and adoption of community-backed standards for effective reporting of AI applications. AI practitioners should also define use space of a model, and evaluate resource credibility using, e.g., these Ten Simple Rules^61^. It is also good practice to use well-known metrics to quantify the performance, reliability, reproducibility, and statistical soundness of AI predictions.

Current trends in explainable AI include the integration of domain knowledge in the design of AI architectures, training and optimization schemes, while also leaving room for serendipitous discovery^62,63^. At the end of the day we expect AI to shed light on novel features and patterns hidden in experimental datasets that current theories or phenomenology have not been able to predict or elucidate^64^. Exploring foundation AI models, such as GPT-4^65^, provides new insights on what the model has learned, and helps understand concepts such as model memorization and deep generalization.

### Holy grail of FAIR science

We identified the following objectives and end-goals of FAIR initiatives.

- As stated before, FAIR is not the end-goal. It is a journey of improving practices and adapting research resources along with technology innovations. FAIR contributes by enabling discovery and innovation. It will also help us identify best practices that lead to sustainability, lasting impact, and funding.
- Software, datasets, and AI models are all first class research objects. Investments and participation in FAIR activities should be considered for career advancement, tenure decisions, etc.
- Since digital assets cannot be open source forever (indefinite funding), FAIR initiatives should also inform what data, AI models and other digital assets should be preserved permanently.
- Leverage scientific data infrastructure to automate^66^ the validation and assessment of the novelty and soundness of new AI results published in peer-reviewed publications.
- Create user friendly platforms that link articles with AI models, data, and scientific software to quantify the FAIRness of AI models, e.g., the Physiome Project (https://journal.physiomeproject.org/), the Center for Reproducible Biomedical Modeling (https://reproduciblebiomodels.org), and the Garden project.
- Recent approaches have showcased how to combine data facilities, computing resources, FAIR AI models, and FAIR and AI-ready data to enable automated, AI-driven discovery^8^.

Creating FAIR discovery platforms for specific disciplines can possibly lead to silos, which would cut short the expected impact of FAIR initiatives. Therefore, synergies among ongoing efforts are critical to link AI model repositories, data facilities, and computing resources. This approach will empower researchers to explore and select available data and AI models. Following clear guidelines to publish and share these digital assets will facilitate the ranking of AI models according to their performance, ease of use and reproducibility; and for datasets according to their readiness for AI R&D and compatibility with modern computing environments. This approach is at the heart of the Garden Project, which will deliver a platform in which FAIR AI models for materials science, physics, and chemistry are linked to FAIR data, and published in a format that streamlines their use on the cloud, supercomputing platforms or personal computers. AI Model Gardens will enable researchers to cross-pollinate novel methods and approaches utilized in seemingly disconnected disciplines to tackle similar challenges, such as classification, regression, denoising, forecasting, etc. As these approaches mature, and researchers adopt FAIR principles to produce AI-ready datasets, it will be possible to identify general purpose AI models, paving the way for the creation of *foundation AI models*, which are trained with broad datasets and may then be used for many downstream applications with relative ease^67–69^. An exemplar of this approach in the context of materials science was presented by Hatakeyama-Sato and Oyaizu^70^, in which an AI model was trained with diverse sources of information, including text, chemical structures, and more than 40 material properties. Through multitask and multimodal learning, this AI model was able to predict 40 parameters simultaneously, including numeric properties, chemical structures, and text.

Achieving the expected outcomes of FAIR initiatives requires coordinated scientific exploration and discovery across groups, institutions, funding agencies and industry. The Bridge2AI program is an example that such interdisciplinary, and multi-funding agency approach is indeed possible. Well defined, targeted efforts of this nature will have a profound impact in the practice of AI in science, engineering and industry, facilitating the cross-pollination of expertise, knowledge and tools. We expect that this document sparks conversations among scientists, engineers and industry stakeholders engaged in FAIR research, and helps define, implement and adopt an agreed-upon, practical, domain-agnostic FAIR framework for AI models and datasets that guides the development of scientific data infrastructure and computing approaches that are needed to enable and sustain discovery and innovation.
